# Optimization of Complete Rat Heart Decellularization Using Artificial Neural Networks

**DOI:** 10.3390/mi13010079

**Published:** 2022-01-02

**Authors:** Greta Ionela Barbulescu, Taddeus Paul Buica, Iacob Daniel Goje, Florina Maria Bojin, Valentin Laurentiu Ordodi, Gheorghe Emilian Olteanu, Rodica Elena Heredea, Virgil Paunescu

**Affiliations:** 1 Immuno-Physiology and Biotechnologies Center (CIFBIOTEH), Department of Functional Sciences, “Victor Babes” University of Medicine and Pharmacy, No. 2 Eftimie Murgu Square, 300041 Timisoara, Romania; florinabojin@umft.ro (F.M.B.); vpaunescu@umft.ro (V.P.); 2 Department of Clinical Practical Skills, “Victor Babes” University of Medicine and Pharmacy, No. 2 Eftimie Murgu Square, 300041 Timisoara, Romania; elena-rodica.heredea@umft.ro; 3 Center for Gene and Cellular Therapies in the Treatment of Cancer Timisoara-OncoGen, Clinical Emergency County Hospital “Pius Brinzeu” Timisoara, No. 156 Liviu Rebreanu, 300723 Timisoara, Romania; taddeus.b90@gmail.com (T.P.B.); valentin.ordodi@upt.ro (V.L.O.); 4 Department of Medical Semiology I, “Victor Babes” University of Medicine and Pharmacy, No. 2 Eftimie Murgu Square, 300041 Timisoara, Romania; 5 Advanced Cardiology and Hemostaseology Research Center, “Victor Babes” University of Medicine and Pharmacy, No. 2 Eftimie Murgu Square, 300041 Timisoara, Romania; 6 Department of Applied Chemistry, Organic and Natural Compounds Engineering, Faculty of Industrial Chemistry and Environmental Engineering, “Politehnica” University Timisoara, No. 2 Victoriei Square, 300006 Timisoara, Romania; 7 Department of Pathology, “Dr Victor Babes” Clinical Hospital of Infectious Disease and Pneumophysiology, 300041 Timisoara, Romania; olteanu.gheorghe@umft.ro; 8 Department of Pathology, “Louis Turcanu” Children’s Clinical Emergency Hospital, 300041 Timisoara, Romania

**Keywords:** regenerative medicine, tissue engineering, decellularized extracellular matrix, machine learning, deep convolutional neural networks

## Abstract

Whole organ decellularization techniques have facilitated the fabrication of extracellular matrices (ECMs) for engineering new organs. Unfortunately, there is no objective gold standard evaluation of the scaffold without applying a destructive method such as histological analysis or DNA removal quantification of the dry tissue. Our proposal is a software application using deep convolutional neural networks (DCNN) to distinguish between different stages of decellularization, determining the exact moment of completion. Hearts from male Sprague Dawley rats (n = 10) were decellularized using 1% sodium dodecyl sulfate (SDS) in a modified Langendorff device in the presence of an alternating rectangular electric field. Spectrophotometric measurements of deoxyribonucleic acid (DNA) and total proteins concentration from the decellularization solution were taken every 30 min. A monitoring system supervised the sessions, collecting a large number of photos saved in corresponding folders. This system aimed to prove a strong correlation between the data gathered by spectrophotometry and the state of the heart that could be visualized with an OpenCV-based spectrometer. A decellularization completion metric was built using a DCNN based classifier model trained using an image set comprising thousands of photos. Optimizing the decellularization process using a machine learning approach launches exponential progress in tissue bioengineering research.

## 1. Introduction

Cardiovascular diseases continue to be a challenge in medicine, being the leading cause of death worldwide [1]. Although heart transplantation remains the gold standard treatment, organ donation is limited by the poor number of available replacements [2]. Moreover, complications such as immune rejection, drug-induced side effects because of chronic immunosuppression negatively interfere with the lifetime of the surgical substitution [3]. Immune rejection after transplantation is caused by antigens which initiate an immune response by the host, causing graft failure and recipient death [4].

Researchers in cardiovascular tissue engineering started to provide novel solutions for end-stage heart diseases to address these limitations. Some focus on increasing the availability of organs, while others on reducing the immune response to donor hearts. These complementary concepts guided the development of decellularization techniques. Decellularization is a process that removes all the cellular components of an organ, creating a stable, biologically active scaffold with a functional vasculature system. Besides removing cells, decellularization significantly reduces the immunogenicity of grafts, creating a perfect matrix that subsequently can be seeded with selected progenitor cell populations [5].

More than a decade ago, Ott et al., decellularized the first rodent heart obtaining an extracellular matrix (ECM) that maintained the organ’s three-dimensional (3D) geometry, guiding later repopulation with stem cells. Rat hearts were decellularized by coronary perfusion on a modified Langendorff apparatus using different detergent solutions such as sodium dodecyl sulfate (SDS), polyethylene glycol (PEG), and Triton X-100 [6].

The Langendorff technique of isolated heart perfusion is still extensively used by cardiovascular researchers nowadays. Retrograde cannulation of the aorta delivers the entire perfusate in the coronary circulation by closing the aortic valve’s leaflets. The Langendorff perfused heart model remains an irreplaceable decellularization method. A constant perfusion flow carries the cellular components outside the heart during decellularization, obtaining an acellular matrix at the end of the process [7].

Since the pioneering work of Ott et al., many researchers have tried perfusion decellularization of the heart by chemical [8,9], enzymatic [10], or physical [11,12] means. The chemical decellularization method proved to be more efficient than others, but it has to be used at a low concentration to avoid undesired alterations of the ECM. The use of SDS gave the best results for the complete removal of cells [6]. For larger-sized hearts, such as porcine, the most effective decellularization method seems to be the combination of SDS and Triton X-100 [13].

There are some limitations to overcome when determining successful decellularization. Most research studies define successful decellularization when the deoxyribonucleic acid (DNA) content remains less than 50 ng/mg dry tissue weight. Different staining techniques such as hematoxylin and eosin (H&E) and Masson’s trichrome show clearance of nuclear remnants and preserved collagen in decellularized scaffolds. DAPI, as a valuable stain for nuclear quantitation, visualizes a complete absence of genetic material in the fixed tissue [14,15,16].

Unfortunately, all the criteria for validating decellularization listed above involve damage to the organ. In a previous study, we described a method for characterization of the ECM by determining the concentration of DNA and proteins in the perfusion solution. In brief, the study analyzed the release of DNA and proteins from the heart during decellularization. The assumption was that when the concentration of the analytes became constant, the process was complete as no additional cellular material was being released [17,18]. This method of validating decellularization is harmless for the organ and allows later recellularization. The only disadvantage of using this method for proving complete decellularization is that it requires supervision and collecting samples every 30 min.

To sum up, all that was stated above underline the lack of non-destructive methods for characterizing decellularization. Our research group designed a monitoring system that identifies the exact moment of complete decellularization. An optical profile built using machine learning approach proved to be strongly correlated with the data gathered from the spectrophotometric measurement of DNA/proteins concentration in the decellularization solution and the histological analysis. This automated system aims to identify the moment of complete decellularization, eliminating the collection of samples or the post-procedure organ’s damage.

## 2. Materials and Methods

### 2.1. Heart Explantation

**Figure 1:**
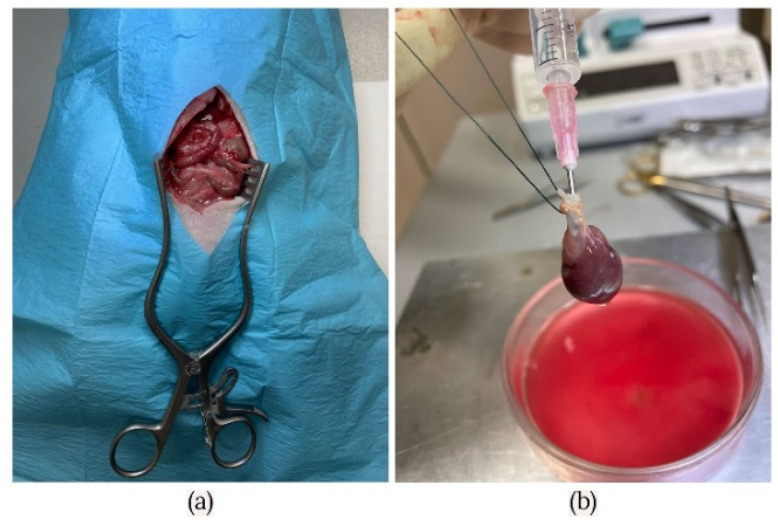
(**a**) Heart explantation—preparing the access to the retroperitoneum. (**b**) Representative image of rat heart before the decellularization process.

Heart extracellular matrices were derived from ten male Sprague Dawley rats of 250–350 g (12–16 weeks old). The hearts had their weight measured on a precision scale (Intelligent-Lab™ HT-84 Analytical Balance). The average weight heart dissected from the rats was 1.28 ± 0.12 g. All animals used in the generation of heart scaffolds were anesthetized with sevoflurane (AbbVie Deutschland GmbH & Co. KG, Wiesbaden, Germany). The protocol included general anesthesia starting with induction at 8% and maintenance at 3.5%. Animals were accordingly prepared to allow sterile surgical access. Pilosity was removed from the entire abdominal–thoracic area and continued with the skin and muscular layer incision. Access to the retroperitoneum was gained by shifting aside the intestine package. Subsequently, the inferior vena cava was exposed and, systemic heparinization was performed using unfractionated heparin (Pan Pharma GmbH, La Selle-en-Luitré, France) at 3.0 IU/g. Median sternotomy allowed access to the mediastinum. Before the heart explantation, the superior and inferior vena cava, the pulmonary veins, and the pulmonary artery were transected. Further, the heart and the ascending aorta were removed from the chest and washed with heparinized saline solution. The organ was attached to the Langendorff device using a 3-0 silk surgical suture. The needle used for the cannulation was inserted less than 1 cm, not too low as it could lead to occlusion of coronary ostium or even to the destruction of aortic valves, compromising the experiment (Figure 1a,b).

### 2.2. Decellularization of Rat Heart

**Figure 2:**
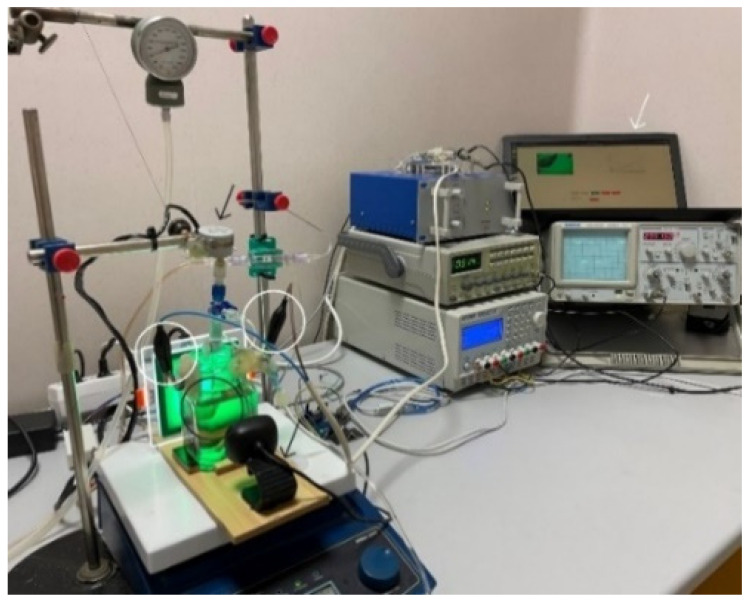
The heart is attached to the Langendorff device using aortic cannulation (black circle). Perfusion is possible with a peristaltic pump, recirculating the 1% SDS solution. A rectangular electric signal is applied to the electrodes from the chamber where the heart is located (white circles). The decellularization process is recorded with a Web camera while a stepper motor moves the heart in the electrochemical cell (black arrows). A deep convolutional neural network (DCNN) model and application were developed to determine the state of the heart during decellularization. The record session view of the application can be seen on the laptop (white arrow).

Heart decellularization creates acellular myocardial scaffolds that perfectly mimic the native architecture, challenging to achieve with synthetic materials. Rat hearts were decellularized as described in our previous study [17]. The protocol provided an alternative electric field system, apparatus, and method of use to obtain decellularized hearts. In brief, all experiments were conducted using a simplified Langendorff device and a decellularization solution containing 1% sodium dodecyl sulfate (SDS; Thermo Scientific, Waltham, MA, USA) in deionized water. After the fixation of the heart into the device, heparinized Krebs solution (Krebs-Ringer Bicarbonate Buffer, Sigma-Aldrich, St. Louis, MO, USA) was perfused for 15 min, so that blood residues are removed from vessels and cardiac chambers. Used Krebs solution was not recirculated within the system. The heart was placed in a decellularization chamber made of glass which contained the 1% SDS solution homogenized during the experiments using a magnetic stirrer (WiseStir MSH-20D hotplate stirrer). The decellularization solution was aspirated from the chamber using a peristaltic pump and reintroduced into the heart via the cannula. The pressure in the decellularization system was about 80 mmHg. This specifically developed protocol generated rectangular electric current (100 mA) with constant frequency (20 kHz), allowing complete decellularization. Compared with standard decellularization methods, this optimized protocol required a lower detergent concentration and a faster treatment time for an optimal whole-heart scaffold (Figure 2).

### 2.3. Spectrophotometric Assay

A spectrophotometric measurement of deoxyribonucleic acid (DNA) and total proteins concentration from the decellularization solution was performed during the experiments. A quasilinear increase of the two parameters was expected, followed by a plateau as the cells were progressively washed out, completing the process. In practice, 10 µL samples were taken from the solution tank at a 30 min interval using an automatic micropipette. We used a small volume spectrophotometer for the measurements (NanoDrop 1000 Spectrophotometer, Thermo Scientific, Waltham, MA, USA). All results obtained by this method were reported normalized to the unit mass of the hearts.

### 2.4. The Monitoring System

**Figure 3:**
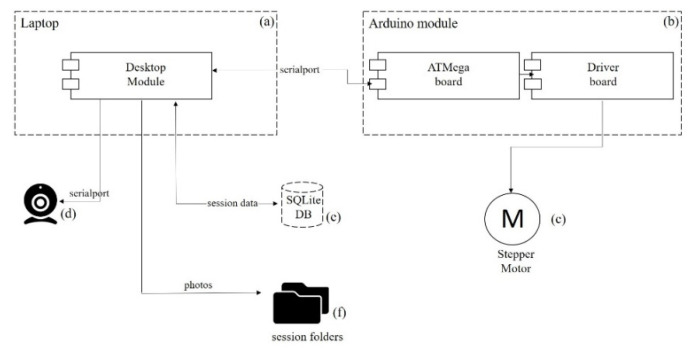
The Monitoring System is composed of the following elements: (**a**) Desktop module, (**b**) Arduino module, (**c**) Stepper motor, (**d**) Web Camera, (**e**) Database, and (**f**) Session folders.

The Monitoring System aims to prove a strong correlation between the data gathered from periodically sampling the decellularization solution and the state of the heart that can be visualized with an OpenCV-based spectrometer (Figure 3).

The Desktop module (Figure 3a) is the leading component in the system, controlling the rest of the elements and coordinating the monitoring process. We call each decellularization a session, containing information about an experiment from beginning to end, meaning all the photos and metrics collected during a recording. Every decellularization session is recorded with a Web camera while a stepper motor moves the heart in the electrochemical cell (Figure 2). The Desktop module sends commands to the Arduino module (Figure 3b) and moves the stepper motor (Figure 3c) 180° clockwise and counter-clockwise from the starting point, completing a full 360° rotation of 400 steps. After every step, one photo is taken, saved to the file system, and processed. The 360° rotation completes a cycle. A cycle contains 400 photos shot with the Web camera (Microsoft LifeCam HD-3000; Figure 3d) connected to the Desktop module. All information is stored in the database (Figure 3e), and every decellularization session has its photos in a separate folder (Figure 3f).

The Desktop module had multiple functionalities implemented. Monitoring every session and saving experimental data could be established. Comparing sessions and access to session history was also possible. Reprocessing older data with newer versions of the application allowed us to make constant updates.

**Figure 4:**
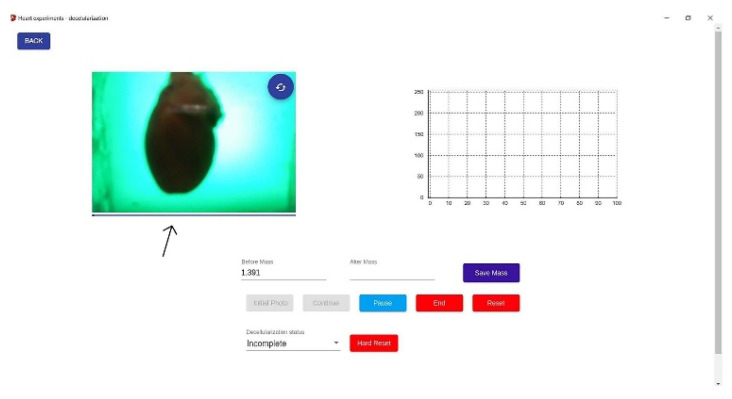
Application programming Interface. Once the process begins, the graphic of the live spectrometer metric can be seen on the upper right side. The progress bar below the camera stream is the classification metric (built with a Deep Convolutional Neural Network model) that shows the progress of the decellularization process (black arrow). The buttons below control the session and allow different commands such as starting, pausing, and ending. Resetting the position of the stepper motor to the initial phase is also possible. The weight of each rat heart is introduced in the program before starting the decellularization process.

The record session view of the application is very suggestive and easy to use. It allows researchers to view the video stream of the webcam that the application is working with (Figure 4).

### 2.5. Collection of Data and Spectrometric Assay

**Figure 5:**
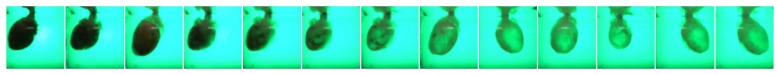
Serial photos were collected from the beginning to the end of the decellularization process. The macroscopic images of the rat heart show the myocardial tissue’s changes. The very end moment of decellularization could not be settled with the human eye, being impossible to pick the differences between the last photos.

Images were saved in the corresponding session folder, classified from 0% to 100% (Figure 5). The spectrometer service processed each image, obtaining a spectrometer metric by extracting the heart pixels. The mean value was calculated for each cycle because of the large number of images acquired. These metrics were stored in the database along with session-related data. It was established that when the visual appearance of the heart stops changing, the metric reaches a plateau, meaning the heart is decellularized, and the process is complete. The decellularization times obtained were also normalized to the unit weight of each heart involved in the experiments.

### 2.6. Training and Testing of Neural Networks

Deep learning is a form of machine learning technique based on artificial neural networks (ANN). It is capable of learning from large amounts of complex, unorganized data. Deep convolutional neural networks (DCNN) are most commonly used to identify patterns in images, focusing on object detection and image classification [19,20,21,22].

**Figure 6:**
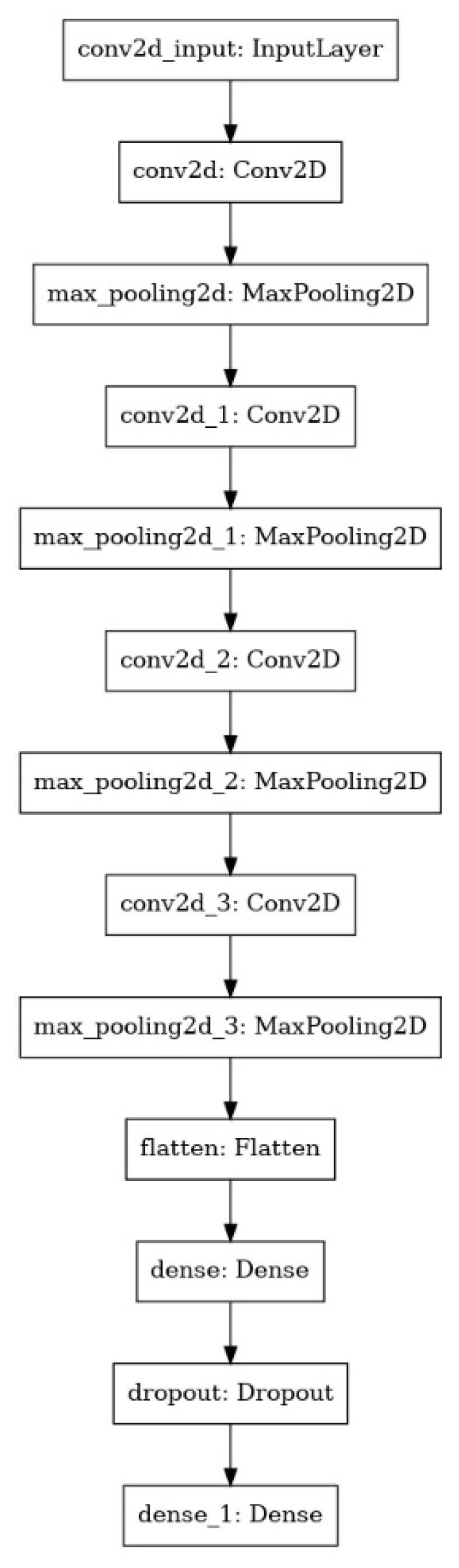
The DCNN model’s architecture.

The classification service was built with a DCNN model trained to classify the degree of completion of the images collected during the decellularization process (Figure 6). A decellularization session contained up to 50,000 collected images with 1280 × 720 pixels resolution. Raw images collected were of this resolution. However, the heart area was cropped, removing unnecessary pixels and resizing the image to 200 × 200 resolution to reduce the resources needed to train a model on such a dataset.

After designing the spectrometer metric and detecting its plateau, we split the image dataset into 11 collections (equal segments of the session parts). The last collection contained images from the end of the session, where the spectrometer metric reached the plateau.

The DCNN was trained on 72,417 images using image augmentation to help generalize training with this dataset. Validation was achieved on 9061 images that were not used during training. Testing was performed using 9060 images that the model never met during training and validation. This process resulted in a model with 95% accuracy.

The metric was calculated using the classification result from each cycle by making a rolling window mean. Our theory was that every metric should start at 0% and end at 100% if complete decellularization. This metric showed a progress bar in the record session view to let researchers know when the process was complete (Figure 4—black arrow).

### 2.7. Histological Examination of the Whole Decellularized Heart

When the software application considered the decellularization complete, the hearts were fixed in 4% buffered formaldehyde solution (Roti-Histofix, Carl Roth GmbH, Karlsruhe, Germany), paraffin-embedded, and sectioned. Three standardized regions were analyzed: apical, mid-ventricular, and basis-near region. After rehydration, sections were first stained with hematoxylin and eosin (H&E, Sigma-Aldrich, St. Louis, MO, USA; Merck & Co., Kenilworth, NJ, USA). This principle tissue stain colors basophilic structures in blue (cell nuclei) and eosinophilic in pink (extracellular matrix and cytoplasm). In order to validate the protocols’ decellularization efficacy and the integrity of the remaining scaffold, Masson’s trichrome (Bio-Optica, Milano, Italy) staining techniques were also used for visualization of collagenous connective tissue fibers. The results were visualized using a light microscope (Leica DM750 Clinical Microscope, Leica Microsystems, Wetzlar, Germany) and a Panthera L Microscope (Motic, Richmond, CA, USA).

## 3. Results

### 3.1. Whole-Heart Decellularization

**Figure 7:**
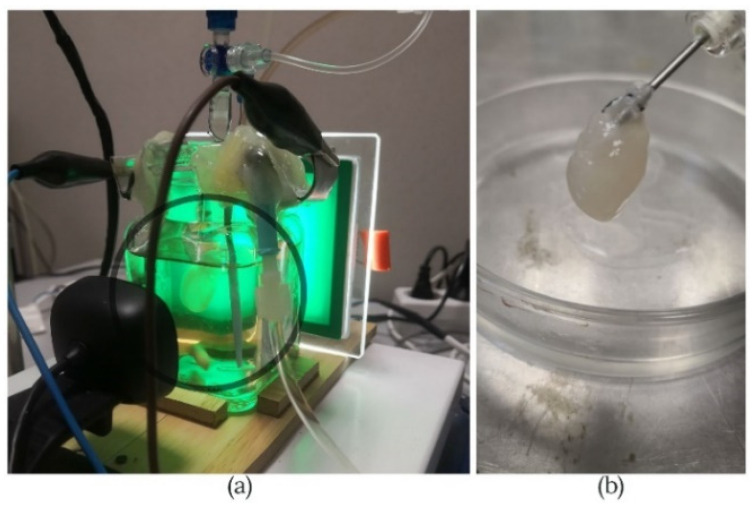
(**a**) Rat heart floating in decellularization solution in the perfusion chamber (black circle). (**b**) The macroscopic aspect of the rat heart ECM.

Rat hearts (*n* = 10) were decellularized and analyzed using the alternative electric field system briefly described in the previous section. The scaffolds were macroscopically examined for structural integrity and preservation of native ECM. All hearts became translucent, with no visible tissue remnants, meaning that all the cells had been washed out. The decellularization protocol preserved the 3D geometry of the cardiac chambers, valvular architecture, and the coronary vascular system (Figure 7a,b).

### 3.2. DNA/Proteins Quantification

**Figure 8:**
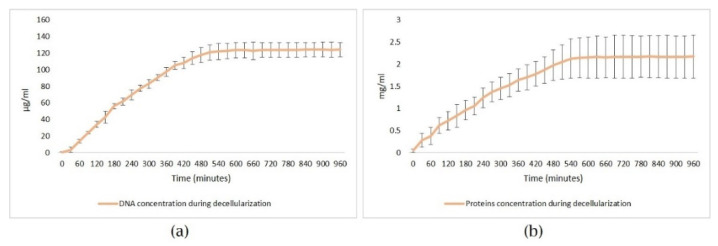
The mean value from the 10 decellularization sessions of total (**a**) DNA and (**b**) proteins concentration measured in the decellularization solution. Both DNA and proteins concentration reached the plateau at around 540 min.

The DNA and proteins concentration in the decellularization solution was determined using ultraviolet absorption spectrophotometry. In all cases, the graphical method determined when the concentration of the two analytes reached a stationary value, i.e., the “plateau of the concentration curve”. This moment was determined by a mathematical algorithm designed by us, representing the differences between two consecutive measurements for each analyte. Using this method, we detected when the difference between two successive determinations on the same curve became insignificant (tended to zero). Electric field decellularization with 1% SDS was completed when the concentration of DNA and proteins in the solution remained constant (Figure 8a,b).

### 3.3. Optimization of Rat Heart Decellularization Using a Deep Convolutional Neural Network (DCNN) Model

**Figure 9:**
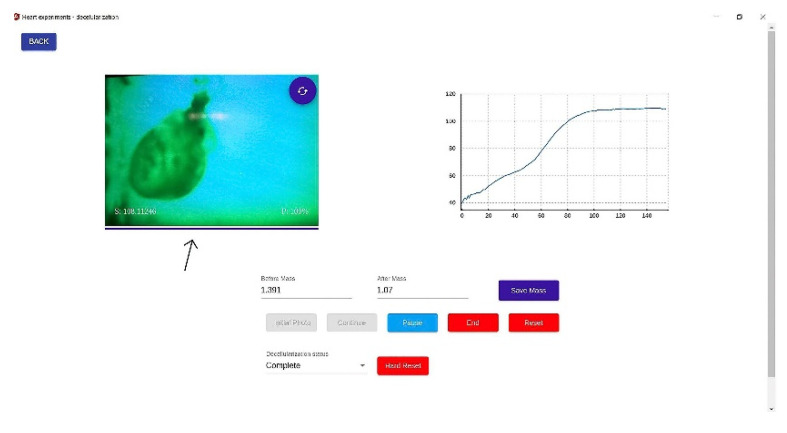
The graphical user interface of a whole decellularized heart. The process is complete when the classification metric represented by the progress bar below the camera is 100%, and the spectrometer metric reaches a plateau. The spectrometer’s graphic shows the number of cycles on the horizontal axis and the mean of the heart pixels per cycle on the vertical axis. As stated above, every cycle has 400 photos. The hearts are being weighed before and after each session, losing 0.32 ± 0.08 g post-decellularization.

The system provided acquisition, processing, and analysis of the rat heart during decellularization. The experimental data made it possible to establish the exact time the organ became translucent, ending the process. On the upper right side of the application, the live spectrometer metric could be seen as a sigmoid curve as the cells were removed from the heart. Reaching the plateau meant completing the process. A classification metric of 100% completed the progress bar below the camera stream, stating complete decellularization (Figure 9).

**Figure 10:**
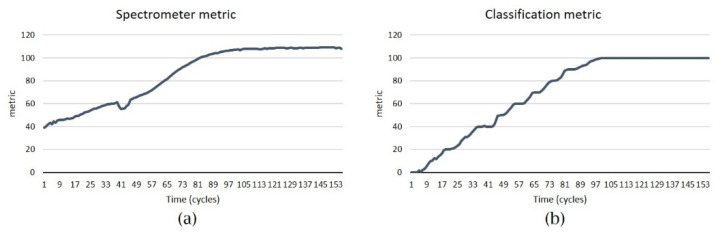
(**a**) Spectrometer metric in time and (**b**) classification metric in time (each point from the horizontal axis is the mean of 400 values collected during a cycle).

Correlating the graphics from the spectrophotometry (Figure 8a,b) with the metrics produced by the spectrometer and classification services (Figure 10a,b), we could state that the model optimized the decellularization process providing an estimation of progress. As a performance measure, the metrics of the classification system for hearts around the same age and weight were precision = 0.95, recall = 0.95, f1-score = 0.95, and accuracy = 0.95.

The spectrometer and the classifier can be used instead of collecting samples from the decellularization solution and can estimate in real-time the decellularization state, thus optimizing the old process.

### 3.4. Microscopic Preservation of Cardiac ECM

**Figure 11:**
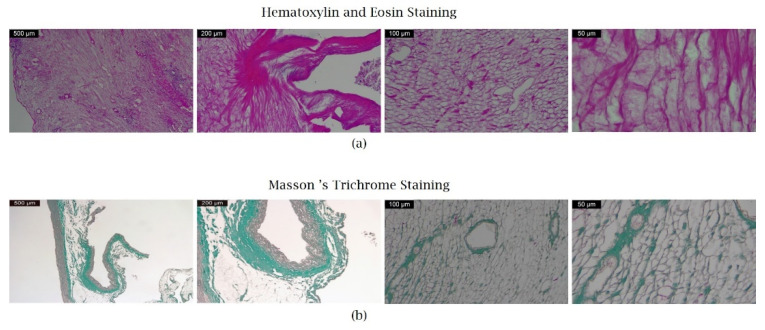
(**a**) Representative H&E staining showing cardiac scaffold of a decellularized whole heart. The ECM remains unaffected. Please note that no nuclei or cellular remnants can be detected (Ob 4×, 10×, 20×, respectively 40×). (**b**) Representative Masson’s Trichrome staining showing preserved collagen and clearance of nuclei (Ob 4×, 10×, 20× respectively 40×).

Two different stainings validated the removal of cellular components and the quality of decellularization. As an initial analytic step, H&E staining showed no intact cells, nuclei, or basophilic structures. Cross-sections of decellularized scaffolds stained with Masson’s trichrome maintained the collagen network but no signs of cardiomyocyte-specific nuclei (Figure 11a,b).

## 4. Discussion

Our study demonstrates, for the first time, the relationship between cell removal in perfusion decellularization and the two metrics produced by an OpenCV-based spectrometer and a Deep Convolutional Neural Network (DCNN) based classifier model. The software application distinguishes between different stages of decellularization, determining through classification the exact moment of completion.

Whole heart decellularization and tissue engineering are expected to revolutionize organ transplantation by eliminating the waiting-list mortality and post-procedure immune rejection [23]. Decellularization of native hearts has made significant progress in the last few years and has experienced an increased level of attention among researchers [24,25].

Rat heart decellularization was performed using an alternating electric field protocol and 1% sodium dodecyl sulfate (SDS) with good results, already described in our previous work. The optimized decellularization method ensures undamaged extracellular matrix (ECM) by applying a low detergent concentration for a short time [17].

Decellularized scaffolds from native organs are essential in tissue engineering if they prove the careful removal of cellular components. ECM integrity means collagen, elastin, and glycosaminoglycans (GAGs) preservation [26].

The quality control of an acellular scaffold has the full attention of researchers due to the lack of standard methods for nondestructive characterization. Histology and scanning electron microscopy (SEM) provide excellent feedback regarding the decellularized scaffold quality, but they require destructive protocols for the samples [27].

Hagen et al. research work used imaging to understand the acellular scaffold’s internal structure. Scaffolds obtained from different organs, such as the esophagus, lung, or liver, were imaged with X-ray phase-contrast computed tomography (PC-CT) as a validation decellularization method. One advantage of this method is that it allows imaging scaffolds prior to and post-transplantation [28]. Another method of quality control of materials for tissue engineering is Raman scattering spectroscopy. It was published by Timchenko et al. in 2017 as a novel assessment of decellularization efficiency using a simple and non-invasive technique [29]. Pereira and co-authors used a non-linear mathematical model using an optical sensor to discriminate between complete and incomplete decellularization. This method proved to be very sensitive, detecting incomplete decellularization even where there was apparent heart transparency [30].

Machine learning approaches started to improve medical care and biomedical research in recent years. It has been used in fields such as radiology, telehealth, clinical care, and even stem cell biology [20,31,32,33].

Given the above, our group developed a software application based on a DCNN model, trained to distinguish between different decellularization stages, establishing the exact completion moment. It is the first computer-aided analysis in decellularization perfusion trained with convolutional neural networks (CNN). Deep learning is becoming an essential computational tool in tissue engineering progress and can meet particular unsolved needs.

## 5. Conclusions

In conclusion, we developed and validated an artificial intelligence-assisted software application to determine through classification when the heart is completely decellularized and, during the process, provide an estimation of progress. Our theory was confirmed by the correlation between the spectrophotometry and the metrics produced by the spectrometer and classification services. The results were promising for hearts of the same age and weight, as the proposed model generalized well on hearts used in the training and validation dataset. For larger hearts, we would need to collect more data. This study could lead to a new standard for heart decellularization, promoting substantial advances in the regenerative medicine field.
